# Solid-State Fermented Okara with *Aspergillus* spp. Improves Lipid Metabolism and High-Fat Diet Induced Obesity

**DOI:** 10.3390/metabo12030198

**Published:** 2022-02-23

**Authors:** Natsumi Ichikawa, Li Shiuan Ng, Saneyuki Makino, Luo Lin Goh, Yun Jia Lim, Ferdinandus, Hiroyuki Sasaki, Shigenobu Shibata, Chi-Lik Ken Lee

**Affiliations:** 1 Laboratory of Physiology and Pharmacology, School of Advanced Science and Engineering, Waseda University, Wakamatsu-cho 2-2, Shinjuku-ku, Tokyo 162-8480, Japan; natsu3@ruri.waseda.jp (N.I.); s.makino@fuji.waseda.jp (S.M.); hiroyuki-sasaki@asagi.waseda.jp (H.S.); 2 Division of Chemistry and Biological Chemistry, School of Physical and Mathematical Sciences, Nanyang Technological University, 21 Nanyang Link, Singapore 637371, Singapore; lishiuan001@e.ntu.edu.sg (L.S.N.); gohl0023@e.ntu.edu.sg (L.L.G.); limy0263@e.ntu.edu.sg (Y.J.L.); fe0003us@e.ntu.edu.sg (F.)

**Keywords:** okara, *Aspergillus oryzae*, *Aspergillus sojae*, solid-state fermentation, anti-obesity

## Abstract

Okara is a major by-product of soymilk and tofu production. Despite retaining abundant nutrients after the process, okara is often under-utilized. In this study, solid-state fermentation (SSF) of okara was carried out using a koji starter (containing both *Aspergillus oryzae* and *Aspergillus sojae*) with the intention of releasing its untapped nutrients. Its effects on lipid metabolism in diet-induced obesity (DIO) were observed. The nutritional profile of fermented okara was elucidated using the following parameters: total phenolic content (TPC), pH, protein content, dietary fiber, amino acid content, and free sugar content. In vivo experiments were conducted using high-fat diets supplemented with unfermented okara and fermented okara over three weeks. Supplementation with fermented okara reduced body weight gain, adipose tissue weight, the serum triglyceride profile, and lipid accumulation in the liver, and altered the mRNA expression levels related to lipid metabolism; however, it did not affect pH and short-chain fatty acid (SCFA) production in this study. In conclusion, high-fat diets supplemented using okara fermented with *Aspergillus* spp. improved the lipid metabolism in mice, due to their high nutritional value, such as TPC, soy protein, and amino acids, and their synergistic effects without altering the gut microbiota.

## 1. Introduction

Okara is the insoluble residual components of ground soybean (*Glycine max*), obtained as a by-product of the soymilk and tofu production process. Asian countries such as China, Korea, and Japan have a high soybean consumption, which results in the generation of large amounts of okara. For instance, ~14 million tons of okara were produced worldwide in 2019 alone [1]. However, most of it is used as animal feedstock, incinerated, or landfilled because of its perishability [1]. Okara has a high moisture content of ~70–80% on wet basis [2], restricting its use for the food industry. There has been research based on the idea of utilizing food waste [3]. Therefore, further research on the potential use of okara is indispensable, both for the environment and the economy.

In recent years, there has been growing interest in incorporating okara into food, owing to its high nutritional value [2,4]. When dried, okara is estimated to be composed of an estimated 50% dietary fiber; 25% protein; 10–20% lipids; and varying amounts of monosaccharides such as glucose, galactose, arabinose, xylose, and uronic acid, as well as polyphenols [1,2,4,5]. Higher substitutions of okara in biscuits have been shown to progressively slow in vitro starch digestion and its subsequent glucose release [5]. Furthermore, incorporating okara in gluten-free cookies increases the protein and fiber content [6], demonstrating the potential of okara as a functional and beneficial ingredient in the human diet.

SSF is a type of fermentative manufacturing process that occurs in the absence of free water. Hence, the substrates used must have enough moisture to support the growth and metabolism of the microbes in the fermentation medium [7]. SSF is traditionally used in the food industries of many Asian countries to produce fermented foods such as miso, soy sauce, and tempeh [8]. In Japan, fermentation starter cultures are called koji starters, which typically consist of steamed rice inoculated with the filamentous fungus *Aspergillus oryzae*. Other fungi such as *A. sojae*, *A. luchuensis*, and *A. awamori* are also used in the food industry, although not as frequently [9]. *Koji* starters can also be mixed cultures of the aforementioned microorganisms.

Previous research on the SSF of okara using various food-grade microorganisms such as *A. oryzae*, *Rhizopus oligosporus*, *Lactiplantibacillus plantarum*, and *Saccharomyces cerevisiae* has shown a significant enhancement of its nutritional profile [10,11,12,13]. An increase in antioxidant activity, soluble dietary fiber (SDF), amino acids, simple sugars, short-chain fatty acids (SCFA), beneficial organic acids, and isoflavone aglycones, as well as a decrease in insoluble dietary fiber (IDF) and some anti-nutrients have been reported after fermentation [10,11,12,13]. Thus, solid-state fermented okara has significant potential as a desirable ingredient in the human diet.

In the past decade, okara has attracted research efforts because of its hypocholesterolemic and hypolipidemic effects [14,15]. Despite the aforementioned benefits of okara in its fermented form, its functionality is still a relatively unexplored area. Nonetheless, the few studies that have been carried out prove its considerable potential as a functional food ingredient. In a previous study, okara fermented with *Eurotium cristatum* showed an antidiabetic potential with a potent α-glucosidase and PTP1B inhibitory activity [16]. Additionally, okara fermented with *A. oryzae* can also attenuate the progression of type 2 diabetes by reducing insulin resistance [17]. However, very few studies have investigated the effects of fermented okara on the management of obesity. Furthermore, *Aspergillus* spp. has a direct health benefit as a probiotic, which is defined as living microorganisms [18].

The World Health Organization (WHO) defines obesity as the excessive accumulation of fat in the body, which may have adverse effects on health [19]. Nearly one-third of the world’s population is overweight or obese [20]. The increase in the prevalence of obesity is likely due to the complex interactions between diet, physical activity, environmental and socioeconomic factors, and genetic predispositions [21]. Furthermore, obesity is a risk factor for the development of several metabolic diseases [15,22] such as cardiovascular disease, type 2 diabetes [22], and cancer [23], making it a looming threat to public health.

This study was designed to quantify the effect of SSF okara (fermented with a mixture of *A. oryzae* and *A. sojae*) on DIO in a rodent model. First, the SSF of okara was optimized in vitro using the mixed *Aspergillus* culture. To determine its nutritional value, the fermented okara was then assessed for its amino acid, sugar content, TPC, protein content, and dietary fiber. To determine the functionality of fermented okara in managing DIO, in vivo tests were conducted, in which changes in body profiles (body weight gain and tissue weight), lipid profiles (cholesterol and triglyceride) in the serum and liver, histological analysis in the liver, and the hepatic lipid metabolism gene expression were evaluated.

## 2. Results

### 2.1. Composition of Okara Fermented with Aspergillus spp.

#### 2.1.1. TPC and pH

**Figure 1:**
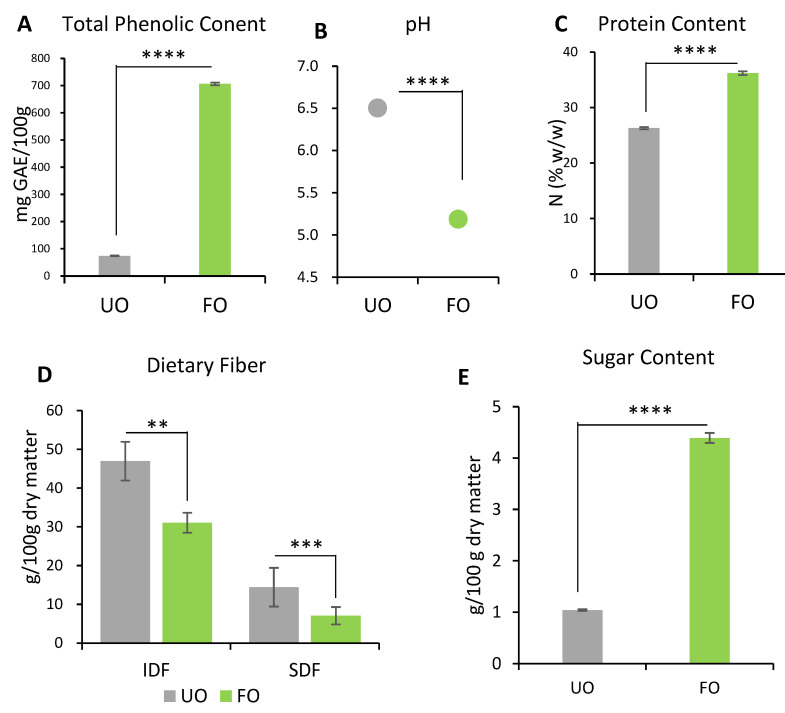
The nutritional profile of unfermented okara (UO) and fermented okara with *Aspergillus* spp. (FO). The TPC (**A**), pH (**B**), protein content (**C**), IDF and SDF (**D**), and total sugar content (**E**) were measured. Data are represented as mean ± SEM (*n* = 3). ** *p* < 0.01, *** *p* < 0.001, and **** *p* < 0.0001 evaluated using Student’s *t*-test.

The TPC was measured (Figure 1A). Compared to unfermented okara, the TPC of fermented okara was significantly higher (*p* < 0.0001).

pH measurements were conducted to investigate the effect of the metabolic products produced during fermentation (Figure 1B). A decrease was observed from 6.50 ± 0.03 to 5.19 ± 0.06 after fermentation (*p* < 0.0001).

#### 2.1.2. Protein Content

The protein content increased after fermentation (Figure 1C), from 26.29 ± 0.21 g/100 g to 36.20 ± 0.34 g/100 g (*p* < 0.0001).

#### 2.1.3. Dietary Fiber

Both IDF and SDF decreased significantly after fermentation (Figure 1D). The IDF decreased by 34% from 46.95 ± 1.30 g/100 g to 31.06 ± 2.23 g/100 g after fermentation (*p* < 0.01). The SDF decreased by 51% from 14.42 ± 0.77 g/100 g to 7.07 ± 0.36 g/100 g after fermentation (*p* < 0.001).

#### 2.1.4. Sugars

In unfermented okara, the large majority of free sugars present were in the form of sucrose at 1.042 ± 0.016 g/100 g (Table 1). After fermentation, sucrose was consumed and converted to glucose, resulting in an increase in free sugars to 4.392 ± 0.098 g/100 g in fermented okara (Figure 1E). Fructose and maltose were not detected in either the unfermented or fermented okara (Table 1).

#### 2.1.5. Amino Acid

The amount of amino acids in okara before and after fermentation and the respective changes are shown in Table 2. Fermentation using *Aspergillus* spp. significantly increased the free amino acids in okara by an average of 24-fold. The majority of the changes observed in amino acids were due to an increase in asparagine, glutamic acid, glutamine, threonine, and serine, with glutamine accounting for 35% of the overall amino acids in fermented okara.

### 2.2. Animal Experiment

#### 2.2.1. Body Profile (Body Weight Gain, Food Intake, and Energy Intake)

Mice were randomly divided into four groups (ND, HD + CON, HD + UO: *n* = 6, HD + FO: *n* = 7; treatments explained below). The groups were fed their respective experimental diets for 3 weeks, namely: normal diet (ND), high-fat diet with 20% control diet (HD + CON), high-fat diet with 20% unfermented okara (HD + UO), and high-fat diet with 20% okara fermented using *Aspergillus* spp. (HD + FO). The composition of the experimental diet is shown in Table 3.

**Figure 2:**
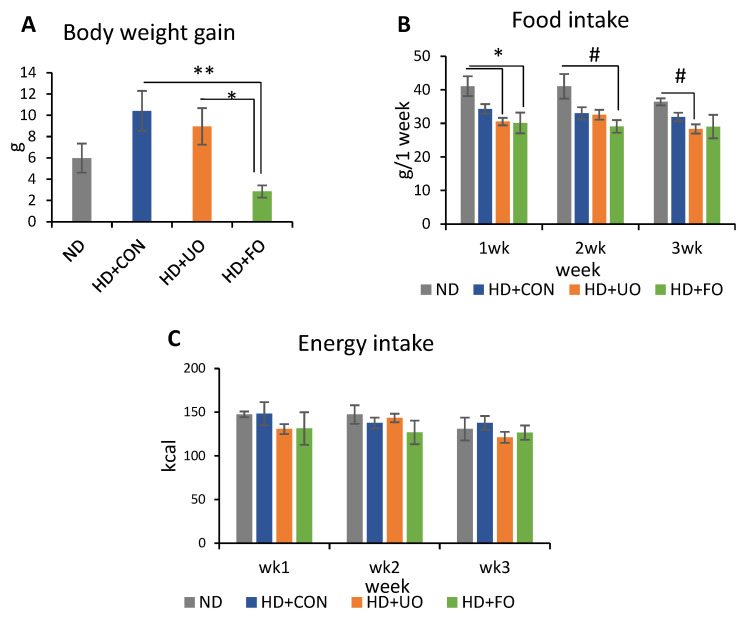
Body weight gain, food intake, and energy intake. Body weight gains (**A**) were differences between body mass in day 0 and day 21. Food intake (**B**) was monitored weekly. Energy intake (**C**) was converted from food intake using calorie shown in Table 3. Data are represented as mean ± SEM (ND, HD + CON, HD + UO: *n* = 6, HD + FO: *n* = 7) * *p* < 0.05, ** *p* < 0.01 evaluated using one-way ANOVA with Tukey’s post hoc test. # *p* < 0.05 evaluated using the Kruskal–Wallis test with Dunn’s post-hoc test.

All body weight gains were reported relative to the body weight on day 0. The body weight gain of the HD + FO-fed mice was significantly lower than that of the HD + CON and HD + UO-fed mice (Figure 2A). The diet intake for all mice groups were measured and converted to caloric intake (Figure 2B). This was carried out as the amount of calories for each diet differed slightly, as highlighted in Section 4.3.1. The intake calories for the mice in all four groups did not significantly differ from one another (Figure 2C).

#### 2.2.2. Tissue Weight (Liver Wight and Adipose Weight)

**Figure 3:**
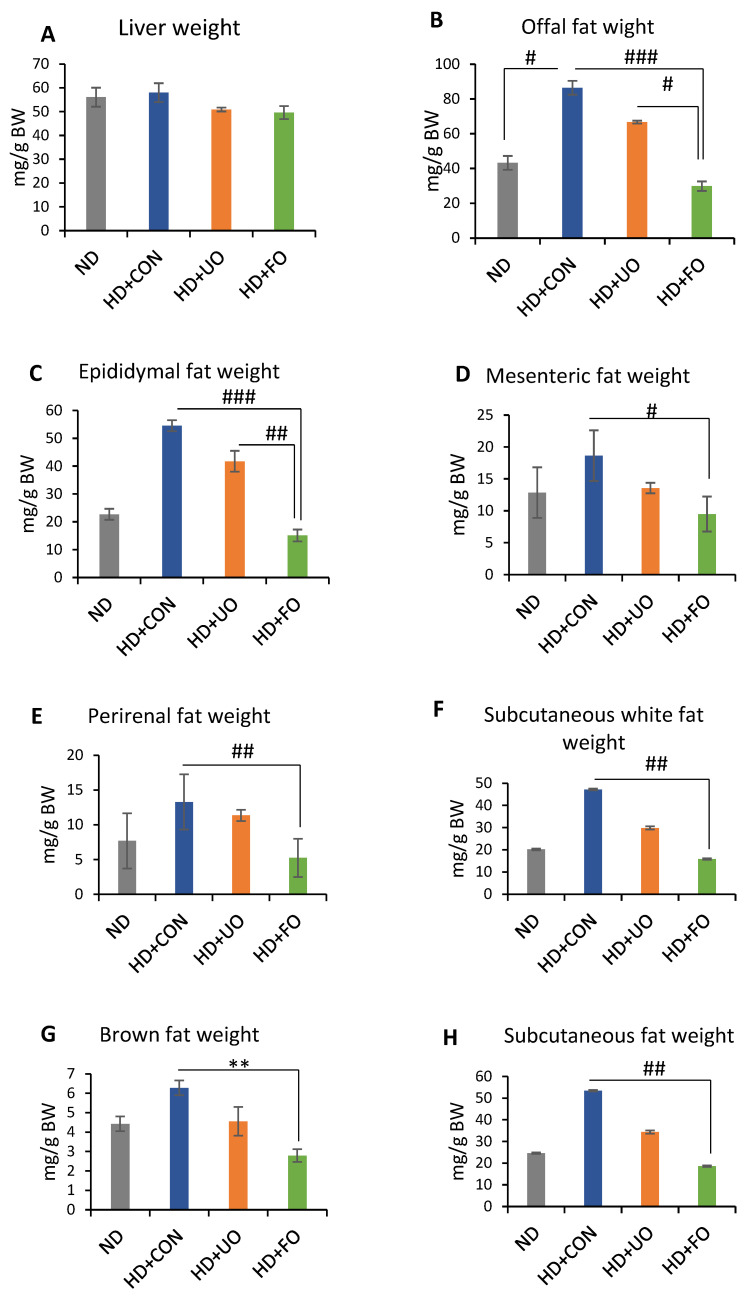
Tissue (liver and adipose) weight corrected by body weight. Liver (**A**), offal fat (**B**), epididymal fat (**C**), mesenteric fat (**D**), perirenal fat (**E**), subcutaneous white (**F**), brown fat (**G**), and subcutaneous (**H**) fat were collected and weighed on Day 21. Data are represented as mean ± SEM (ND, HD + CON, HD + UO: *n* = 6, HD + FO: *n* = 7) ** *p* < 0.01 evaluated using one-way ANOVA with Tukey’s post hoc test. # *p* < 0.05, ## *p* < 0.01, ### *p* < 0.001 evaluated using the Kruskal–Wallis test with Dunn’s post-hoc test.

The liver and the adipose weights were measured to determine the reason for the changes of body weight gain. The tissue weights on day 21 were corrected for body weight. The liver weights did not significantly differ between the four groups (Figure 3A), however, the adipose tissue weights were different depending on supplemented diets (Figure 3B–E). The offal fat weight of HD + FO-fed mice was small compared with the HD + CON and HD + UO-fed mice, and HD + CON-fed mice were smaller than those of ND-fed mice (Figure 3B). A closer look at the offal fat showed that epididymal, mesenteric, and perirenal fat weight of HD + FO-fed mice were lower than those of HD + CON-fed mice (Figure 3C–E). The subcutaneous fat weight of HD + FO-fed mice was lower than that of HD + CON-fed mice, overall and in both brown fat weight and subcutaneous white fat weight (Figure 3F–H).

#### 2.2.3. Lipid Profile in the Serum (Cholesterol, Triglyceride)

**Figure 4:**
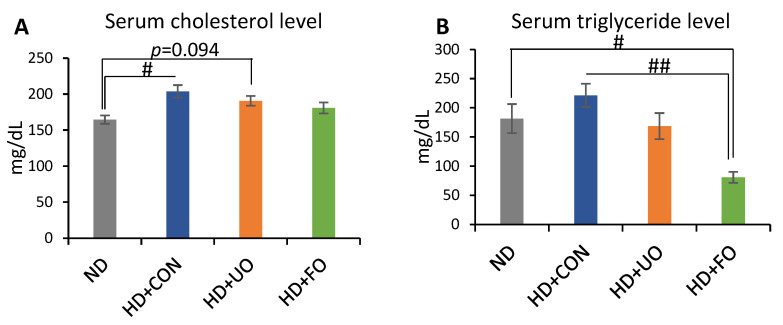
Lipid profile in the serum (cholesterol and triglyceride). Serum cholesterol (**A**) and serum triglyceride (**B**) levels of mice fed each experimental diet for 3 weeks. Data are represented as mean ± SEM (ND, HD + CON, HD + UO: *n* = 6, HD + FO: *n* = 7) # *p* < 0.05, ## *p* < 0.01 evaluated using the Kruskal–Wallis test with Dunn’s post-hoc test.

Lipid profiles in the serum were measured to elucidate the effect of fermented okara with *Aspergillus* spp. on the management of DIO. The serum cholesterol levels were lower in the ND-fed-mice than in the HD + CON-fed mice (Figure 4A). The serum triglyceride levels of the HD + FO-fed mice were lower than those of the ND and HD + CON-fed mice (Figure 4B). Cholesterol levels in the serum, which were elevated by feeding high-fat diets, were suggested to be suppressed by the supplementation of fermented okara. Triglyceride levels in the serum decreased the supplementation of fermented okara, suggesting that feeding fermented okara reduced lipid accumulation in the serum.

#### 2.2.4. Liver Lipid Profile (Cholesterol, Triglyceride, and Histological Analysis)

**Figure 5:**
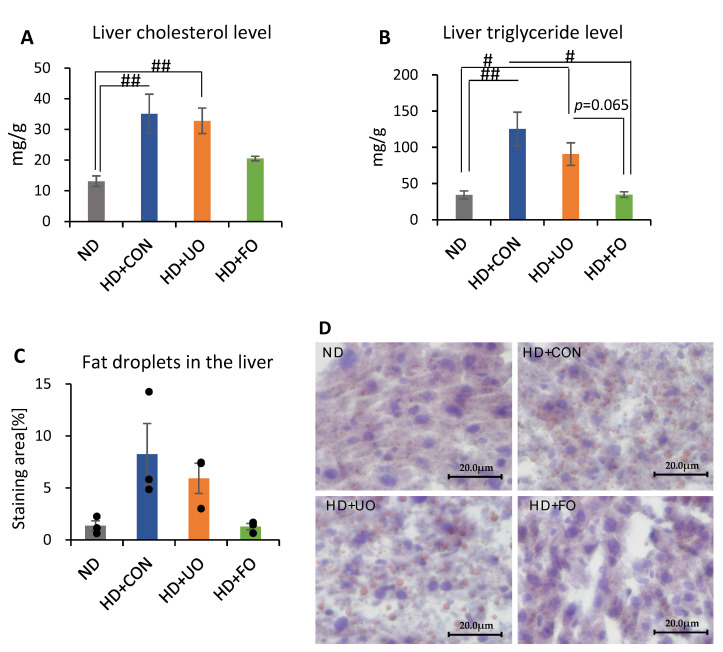
The lipid profile in the liver (cholesterol and triglyceride). Cholesterol lipid (**A**), triglyceride lipid (**B**) levels, lipid droplet size represented by the stained area (*n* = 3; each sample value is shown in black dots) (**C**), Oil Red O staining (**D**) of liver section of mice fed each experimental diet for 3 weeks. Data are represented as mean ± SEM (ND, HD + CON, HD + UO: *n* = 6, HD + FO: *n* = 7) # *p* < 0.05, ## *p* < 0.01 evaluated using the Kruskal–Wallis test with Dunn’s post-hoc test.

The lipid profile in the liver was evaluated using measurements cholesterol and triglyceride, and a histological analysis. The liver cholesterol levels were significantly higher in the HD + CON-fed and HD + UO-fed mice than in the ND-fed mice, however it did not show a significant change between ND-fed mice and HD + FO-fed mice (Figure 5A). The liver triglyceride levels were higher in ND-fed mice than in the HD + CON-fed mice and HD + UO-fed mice (Figure 5B). In addition, the liver triglyceride level was lower in HD + FO-fed mice than in HD + CON-fed mice and HD + UO-fed mice (non-significant; *p* = 0.065). For the histological analysis, the lipid droplets were stained by Oil Red O staining (Figure 5D), and their areas were quantified by image analysis (Figure 5C). Lipid droplets in the liver of ND-fed and HD + FO-fed mice were smaller than those of HD + CON- and HD + UO-fed mice (Figure 5C,D), however there was no significant difference. Feeding a high-fat diet induced fat accumulation in the liver, and the supplementation of fermented okara suppressed fat accumulation. However, the supplementation of unfermented okara did not show a fat-lowering effect.

#### 2.2.5. mRNA Expression Levels of Genes in Liver

**Figure 6:**
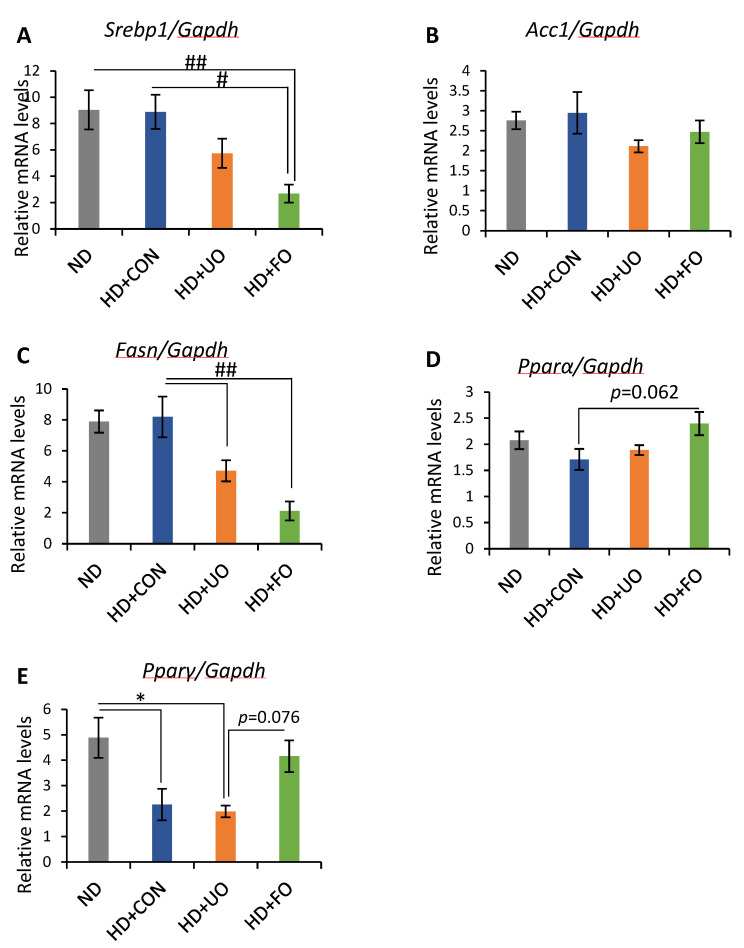
Gene expression levels in the liver. Relative RNA expression levels of fatty acid metabolism-related genes in the livers: *Srebp1* (**A**), *Acc1* (**B**), *Fasn* (**C**), *Pparα* (**D**), and *Pparγ* (**E**). Data are represented as mean ± SEM (ND, HD + CON, HD + UO: *n* = 6, HD + FO: *n* = 7) * *p* < 0.05 evaluated using one-way ANOVA with Tukey’s post hoc test. # *p* < 0.05, ## *p* < 0.01 evaluated using the Kruskal–Wallis test with Dunn’s post-hoc test.

To elucidate the pathways that reduced the triglyceride levels, the mRNA expression levels of *Fasn*, *Acc1*, *Srebp1* (related to fatty acid synthesis), *Pparα*, and *Pparγ* (related to fatty acid metabolism) were measured. The expression levels of *Srebp1* and *Fasn* were significantly lower in the HD + FO-fed mice than in the ND and HD + CON-fed mice (Figure 6A,B). However, the expression levels of *Acc1* were not different between the groups (Figure 6C). The expression levels of *Pparα* and *Pparγ* tended to be higher in HD + FO-fed mice than in HD + CON-fed mice (Figure 6D,E).

## 3. Discussion

In this study, okara fermented for four days using the koji starter SP-01 (a mixture of *A. oryzae* and *A. sojae*) appeared to improve the nutritional factors. The TPC, protein content, free amino acids, and sugars increased, while those of IDF and SDF decreased (Figure 1 and Table 1 and Table 2).

An increase in TPC was observed after fermentation, which is in line with the literature. The fermentation of okara using microorganisms such as *Yarrowia lipolytica* [2] and *Monascus anka* AOK 2026 [24] have been shown to increase TPC levels. According to Vong et al. (2016), the reason for this could be tyrosine catabolism by *Y. lipolytica*, however, the underlying mechanism is not well known [2]. A high correlation between TPC in okara fermented using *Monascus anka* AOK 2026 and antioxidant activity has previously been reported [24], suggesting that okara fermented with *Aspergillus* spp. may have an enhanced antioxidant activity compared to its unfermented counterpart.

Similarly, an increase in protein content was observed after fermentation, which is consistent with the current findings. Chan et al. (2019) reported that the fermentation of okara using *E. cristatum* after 10 days resulted in an increase in protein content from 22.3% to 32.6% [16]. According to Sitanggang et al. (2019), the fermentation of okara using *A. oryzae* resulted in an increase in protein content from 24.0% to 41.1% [13]. The increase in protein content after fermentation was likely caused by two mechanisms: microorganisms that metabolize the substrate to fungal proteins, and an increase in fungal proliferation, along with an increase in fungal cells during solid-state yeast treatment [25]. In addition to an increase in protein content, SSF using *A. oryzae* improved the originally low solubility of okara protein [25].

The reduction of IDF during fermentation is due to the enzymatic activity of *A. oryzae* and *A. sojae*, which breaks down glycosidic linkages. The main components of IDF are hemicellulose and cellulose [26], present in the cell walls of okara. IDF is extremely stable, making it indigestible by the human body. However, both *A. oryzae* and *A. sojae* are filamentous fungi that can excrete cellulolytic enzymes, such as cellulase and hemicellulase, to digest cellulose and hemicellulose, respectively [27]. This has two benefits: fungal growth is supported on lignocellulosic biomasses such as okara, and this makes fermented okara more digestible.

The remarkable increase in overall amino acids after fermentation highlights the strong proteolytic ability of *A. oryzae* and *A. sojae* [26]. Protein isolates in okara can be hydrolyzed into smaller peptides and free amino acids [28,29]. Glutamic acid, which increases after fermentation, is expected to give okara an umami flavor, commonly associated with palatable edible ingredients. Essential amino acids, such as histidine, tryptophan, and phenylalanine, have previously been shown to have antioxidant properties [30,31]. Hence, it is highly probable that the increase in amino acids would improve the flavor and nutrient content of okara, while improving its antioxidant capacity.

The increase in glucose arises from the hydrolysis of disaccharides, such as sucrose and IDF. Okara contains approximately 50% cellulose and hemicellulose [2]. The decrease in IDF for fermented okara with *Aspergillus* spp. aligns with the results of increased glucose levels. The glucose produced can be utilized to maintain the growth, development, and proliferation of fungi and produce other metabolites such as amino acids and organic acids [28].

**Figure A1:**
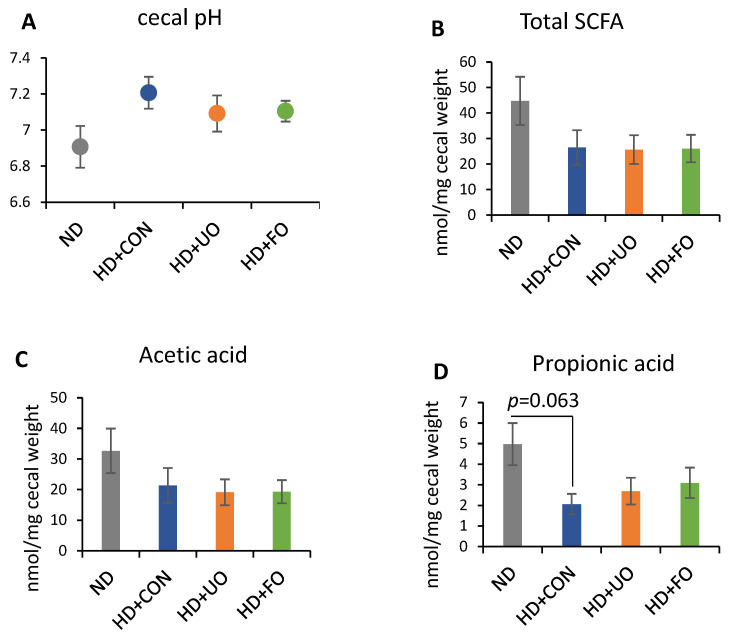

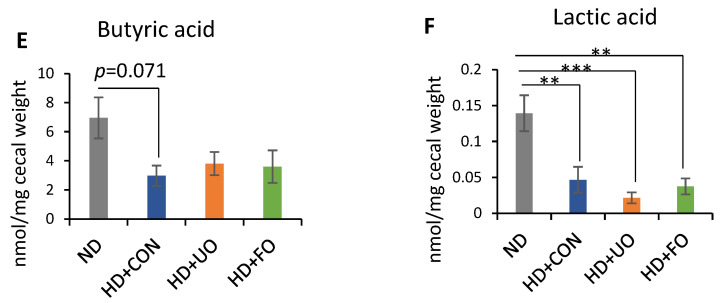
Cecal pH and short chain fatty acid levels. Cecal pH levels (**A**) and the concentration of short chain fatty acid of mice fed each experimental diet for 3 weeks: total SCFA (**B**), acetic acid (**C**), propionic acid (**D**), butyric acid (**E**), and lactic acid (**F**). Data are represented as mean ± SEM (ND, HD + CON, HD + UO: *n* = 6, HD + FO: *n* = 7) ** *p* < 0.01, *** *p* < 0.001 evaluated using one-way ANOVA with Tukey’s post hoc test.

An in vivo experiment showed that the 3-week supplementation of okara fermented with *Aspergillus* spp. improved the lipid metabolism of mice. Body weight gain was suppressed through 3 weeks of feeding with fermented okara (Figure 2A), although the calorie intake did not significantly differ among the four groups (Figure 2C). The differences in some fat tissues showed the same tendency (Figure 3). In addition, the serum and liver triglyceride levels, lipid accumulation in the liver, and gene expression related to lipid synthesis and metabolism in the liver were altered (Figure 4, Figure 5 and Figure 6), suggesting that the TPC, protein content, and amino acids included in fermented okara suppressed lipid synthesis and promoted metabolism. Some studies have reported that dietary fiber can alter the lipolysis process [32], however, the amount of dietary fiber was decreased by fermentation in this study (Figure 1D). In addition, some parameters related to the gut environment, such as caecal pH and SCFA production, did not change between HD + UO and HD + FO-fed mice (Appendix A Figure A1). Therefore, the changes in lipid metabolism caused by fermented okara with *Aspergillus* spp. may be due to nutritional factors that changed during fermentation, rather than prebiotic effects. Nutritional factors, especially TPC [33,34], protein content [35,36], and amino acids [37,38,39], have the potential to suppress diet-induced obesity, hence they may work on lipid synthesis and metabolism in the liver and reduce fat accumulation in the liver. Finally, the triglycerides and cholesterol levels in the blood after being broken down were also reduced.

TPC has beneficial effects in managing obesity through its antioxidant effects. Oxidative stress is both a cause and consequence of obesity, and obesity causes low levels of inflammation and acidic stress [40]. Therefore, obesity and oxidative stress have been studied together, and antioxidants such as polyphenols have shown anti-obesity effects by reducing oxidative stress, lipid synthesis, and accumulation [41]. It is known that okara fermented by *A. oryzae* increases TPC levels. The increase in TPC is strongly correlated with antioxidant effects [2]. The increase in phenolic compounds brought about by fermented okara may help prevent oxidative diseases such as coronary artery disease and cancer. Furthermore, the relationship between natural polyphenols and anti-obesity effects was reported to be due to various mechanisms, including the inhibition of the pancreatic lipase activity [42], promotion of lipolysis [33], prevention of lipogenesis [34], promotion of thermogenesis and lipid metabolism [34], and appetite control [43]. As it has been established that bioactive metabolites in plant extracts, especially phenolic and flavonoid compounds, inhibit pancreatic lipase secreted by the pancreas [44], it is likely that the phenolic compounds in fermented okara also inhibited pancreatic lipase in this study. However, because phenolic compounds are transferred to various tissues after absorption, it is necessary to investigate which specific phenolic components of TPC caused the anti-obesity effect in order to clarify which pathway the phenolic contents in fermented okara caused the anti-obesity effect.

Soy proteins are known to have beneficial effects on lipid metabolism. It has been reported that soy β-conglycinin reduces serum triglyceride levels and improves hepatic lipid metabolism [45]. Some mechanisms for the fat-lowering effect have been proposed: soybean β-conglycinin lowered the activity of *Fasn* and increased the activities of β-oxidation enzymes [35,36]. These changes in mRNA expression levels, which were reported previously, are in agreement with the results of this study. Some studies have concluded that the lipid-lowering effect of soy protein is due to its probiotic effect. However, it has been suggested that the lipid-lowering effect is due to a metabolic pathway modification by β-conglycinin rather than a prebiotic effect, because fermented okara did not improve the gut environment, such as the caecal pH and the production of SCFA.

Amino acids, especially leucine [37,38], branched-chain amino acids [39], and glutamine [46], affect lipid metabolism by regulating lipid metabolism-related genes and lipid profiles in the serum and liver. Amino acids, especially glutamine, which were increased during fermentation, previously improved the lipid metabolism. Glutamine supplementation in rats treated with high-fat diets reduced weight gain and improved insulin action and signaling in the liver and muscle [46]. Therefore, the increase in amino acids during fermentation with *Aspergillus* spp. may have improved the lipid metabolism in this study. However, the results of the present study could not be explained by only amino acids, because some previous research has shown opposite results [47,48] and there are complex pathways regarding the relationship between amino acids and lipid metabolism.

The lipid-lowering effect of okara fermented with *Aspergillus* spp. could be due to the synergistic effect of multiple nutrients rather than each of these nutrients alone. The combination of fibers and phospholipids with soy protein showed a higher hypocholesterolemic effect than soy protein alone [49].

In conclusion, okara fermented with *Aspergillus* spp. showed anti-obesity effects due to some nutritional factors, soy protein, isoflavones, and phospholipids, rather than probiotic effects.

## 4. Materials and Methods

### 4.1. Materials

Okara was collected from Sing Ghee Beancurd Manufacturer (Singapore). The koji starter SP-01 was purchased from Nihon Jyozo Kogyo (Tokyo, Japan). All SP-01 spore suspensions were freshly prepared at a concentration of 106 spores/g (dry weight) of okara. SP-01 consists of both *A. oryzae* and *A. sojae* spores. Both microbes are traditionally used in Japanese fermented products such as soy sauce, sake, and miso.

All mice were purchased from Tokyo Laboratory Animals (Tokyo, Japan). MF and high-fat diets (#D12451) were purchased from Oriental Yeast Co. (Tokyo, Japan) and RESEARCH DIET Inc. (New Brunswick, NJ, USA), respectively.

### 4.2. Fermentation and Characterization

#### 4.2.1. Solid-State Fermentation

After the okara was defrosted and autoclaved, 20 g samples were inoculated with the SP-01 spore suspension in petri dishes; the mixtures were left to ferment in the dark for 4 days at 25 °C. After fermentation, the samples were dehydrated overnight in a 67 °C food dehydrator (Excalibur Food Dehydrators, Sacramento, CA, USA), then they were ground (grinder from Gewürz and Kaffee Mühle, Rommelsbacher, Germany) into particles with sizes of 0.1–0.2 mm. The samples were stored in a refrigerator at 4 °C prior to the subsequent analyses.

#### 4.2.2. Measurement of pH

The samples were dissolved in distilled water, and the pH values were determined using a pH meter (DOCU-pH Meter, Sartorius, Göttingen, Germany).

#### 4.2.3. Determination of TPC

The TPC of the samples were determined using the Folin and Ciocalteu’s phenol (FC) method [50], with minor modifications for the microplate reader. After 100 ± 1 mg of samples were extracted using 50% methanol (*v*/*v*), the samples were centrifuged (15,000 rpm, 10 min, room temperature). The supernatants were diluted 100-fold using distilled water, 1 mL of FC solution (Sigma-Aldrich, St. Louis, MO, USA), and 800 μL of 1M sodium carbonate solution, which were sequentially added to glass vials; these were incubated at 45 °C for 15 min. The absorbance was measured at 760 nm using a UV-VIS spectrophotometer (UV-1800, Shimadzu, Kyoto, Japan). The TPC values were expressed in milligrams of gallic acid equivalents (GAE) per 100 g of dry weight (mg GAE/100 g) and were calculated using a gallic acid calibration curve.

#### 4.2.4. Determination of Protein Content

The Kjeldahl method (AOAC 2001.11) was used to determine the protein content. First, 200 ± 1 mg of the samples, 12 mL of 98% concentrated sulfuric acid, and two catalyst Kjeltabs (FOSS, Hilleroed, Denmark) were added to the Kjeltec test tubes. This was followed by digestion at 420 °C for 1 h using the Kjeltec digestion unit. The tubes were allowed to cool for 15 min before neutralization through the addition of 40% (*w*/*v*) sodium hydroxide in the Kjeltec auto-analyzer unit. The analyzer unit was titrated to a colorimetric endpoint, and the protein content was calculated using a conversion factor of 6.25.

#### 4.2.5. Extraction of Amino Acids and Sugars

First, 250 ± 1 mg of the samples, along with the lactose, norvaline, and sarcosine standards, were extracted with 4 mL 80% ethanol (*v*/*v*) for 30 min at 50 °C while stirring constantly. The suspension was centrifuged (4000 rpm, 20 min, room temperature), and the supernatants were collected. The solvents were removed using a rotary evaporator and were re-dissolved in an ACN/water mixture (2:1, *v*/*v*).

#### 4.2.6. High Performance Liquid Chromatography (HPLC) Analysis of Amino Acids

Amino acid analyses were performed using the Prominence-i series model LC-2030 LT HPLC system from Shimadzu with AdvanceBio AAA column (3.0 mm × 100 mm, 2.7 μm particle size) and a fluorescence detector from Agilent Technologies, Inc. (Santa Clara, CA, USA). The samples were derivatized with FMOC and OPA reagents before injection. Amino acid identification was carried out based on the retention times of the standards, and quantification was performed using five-point calibration curves.

#### 4.2.7. HPLC Analysis of Sugars

Sugar analyses were performed using a Prominence-i series model LC-2030 LT HPLC system from Shimadzu (Kyoto, Japan) with an NH2 column (4.6 mm × 150 mm, 5 μm particle size) and RID-20A refractive index detector from Kromasil (Bohus, Sweden). Sugar identification was carried out based on the retention times of the standards, and quantification was performed using five-point calibration curves.

#### 4.2.8. Determination of Dietary Fibre

The quantities of IDF and SDF were determined according to the AOAC method (991.43) using Megazyme’s total dietary fiber assay kit (Megazyme, Wicklow, Ireland). The samples were enzymatically and sequentially treated (in duplicates) with heat-stable α-amylase, protease, and amyloglucosidase. The residues included IDF and SDF; IDF was isolated by centrifugation. SDF was isolated by precipitation using warm 95% ethanol (*v*/*v*). After both the IDF and SDF residues were washed and dried, the weights of IDF and SDF were calculated by subtracting the respective ash weight and crude protein weight.

### 4.3. Animal Experiments and Profiling

#### 4.3.1. Animals and Experimental Diets

Twenty five 8-week-old male ICR mice were randomly divided into four groups (ND, HD + CON, HD + UO: *n* = 6, HD + FO: *n* = 7; treatments explained below). The groups were fed their respective experimental diets and water, namely: normal diet (ND), high-fat diet with 20% control diet (HD + CON), high-fat diet with 20% unfermented okara (HD + UO), and high-fat diet with 20% okara fermented using *Aspergillus* spp. (HD + FO). The mice were fed their respective diets for 1 week to acclimatize them, after which they were kept under experimental conditions for three weeks. Food intake and body weight were measured weekly. The mice were individually housed in plastic cases under 12 h light/dark conditions, with lights-on time defined as zeitgeber time 0 (ZT0) and lights-off time as zeitgeber time 12 (ZT12). The housing room was maintained at a temperature of 22 ± 2 °C, humidity of 60 ± 5%, and an ambient light intensity of 100–150 lux. All of the animal studies were carried out with the permit number: 2021-A049.

The nutritional composition and total calories of the unfermented okara and fermented okara used in the diets are shown in Table 4. The ingredients of the aforementioned diets are shown in Table 3. The ND was based on an MF. Starch, casein, and corn oil were added to HD + CON to match the nutrient content (carbohydrates, protein, and fat) and caloric content of okara. HD + CON, HD + UO, and HD + FO diets were modified from the #D12451 diet.

After the experimental period, the mice were anesthetized with isoflurane and sacrificed at ZT12. Adipose tissue and liver weights were measured in each mouse. The blood and liver samples were collected and stored at −80 °C for further analysis.

#### 4.3.2. Liver Fat Extraction

The Folch method was used for the extraction of liver fat. To 440 μL homogenate of pre-weighed liver sections, 1100 μL of methanol/chloroform solution (1:2) was added. After vortex, the organic layer was separated by centrifugation at 15,000 rpm for 5 min. Transfer 400 μL of the lower organic layer to a new tube, and dry on a heat block at 80 °C for 10 min. The remaining lipids were dissolved in 200 μL of isopropanol.

#### 4.3.3. Measurement of Lipid Profile in the Serum and Liver

The lipid profile levels in the serum and liver were determined using a commercially available kit (FUJIFILM Wako Pure Chemical Co., Osaka, Japan). The assay was performed according to the manufacturer’s instructions.

#### 4.3.4. Histology Examination

For the histopathological analysis, we used Oil Red O staining using the Oil Red O stain kit (Polysciences Inc., Warrington, PA, USA) based on previous studies. Liver samples were excised and embedded in an OCT compound (Sakura Finetek Japan Co., Osaka, Japan), and serially sectioned at 10 μm. The slides were immersed in 10% formalin for about 10 min, propylene glycol solution for 3 min, Oil Red O solution for 10 min, and 85% propylene glycol solution for 4 min in sequence. After rinsing with deionized water for 2 min, the specimens were stained with hematoxylin solution for 40 s. They were washed with tap water again for 3 min, and kept in deionized water, and finally sealed with Aqua-Poly/Mount (Polysciences Inc., Warrington, PA, USA). All tissue specimens were photographed using a fluorescence microscope (BZ-8100; Keyence Co., Osaka, Japan). The size of the lipid droplets was measured using Image J to quantify the fat accumulation in the liver.

#### 4.3.5. Real-Time Reverse Transcription PCR (RT-PCR)

The relative mRNA expression levels were measured using real-time RT-PCR. The total liver RNA was extracted using RNA-Solv Reagent (Omega Bio-Tek Inc., Norcross, GA, USA). The concentration of each sample was processed to 50 ng/μl with DEPC water using a spectrophotometer (Thermo Fisher Scientific K.K., Tokyo, Japan). The RNA was reverse-transcribed and amplified using the One-Step SYBR RT-PCR kit (Takara Bio Inc., Shiga, Japan) with primers for target genes on a real-time RT-PCR system (PikoReal, Thermo Fisher Scientific, Waltham, MA, USA). The relative mRNA expression levels of the target genes were corrected using *Gapdh* and were analyzed using the ΔΔCt method.

#### 4.3.6. Statistical Analysis

All data are shown as the mean ± standard error of the mean and were analyzed using GraphPad Prism version 9.1.1 (GraphPad Software, San Diego, CA, USA).

For data with two groups, student’s *t*-test was conducted. For data with 3 groups or more, the normal distribution and equal variations were examined using the D’Agostino Pearson/Kolomogorov-Smirnov test and Bartlett’s test, respectively. Parametric test was conducted using a one-way analysis of variance (ANOVA) with Tukey post-hoc test. Non-parametric analysis was conducted using the Kruskal–Wallis test and Dunn’s post-hoc test. The significance level was set at *p* < 0.05.

## Figures and Tables

**Table 1:** Free sugars in okara before and after fermentation. Data represent mean ± SEM of three replicates (*p* < 0.05); ND—not detected, i.e., amount of sugar is lower than LOD. Trace means the amount of sugars is higher than LOD, but lower than LOQ.

	Average (g/100 g Dry Matter)	
Sample/Sugar	UO	FO
Fructose	ND	ND
Glucose	ND	4.392 ± 0.098
Sucrose	1.042 ± 0.016	ND
Maltose	ND	ND
Total sugar	1.042 ± 0.016	4.392 ± 0.098

**Table 2:** Amino acid concentration of okara before and after fermentation. Data represent mean ± standard deviation of three replicates (*p* < 0.05). ND—not detected, i.e., the amount of amino acids lower than LOD.

	Average (mg/100 g Dry Weight)		
Amino Acid	Unfermented Okara	Fermented Okara	Fold Change
Asp	3.221 ± 0.051	58.366 ± 0.798	18
Glu	10.509 ± 0.103	322.036 ± 3.060	31
Asn	0.677 ± 0.014	72.094 ± 0.648	106
Ser	0.847 ± 0.019	47.399 ± 0.540	56
Gln	0.639 ± 0.004	521.395 ± 3.955	816
His/Gly	1.767 ± 0.129	44.09 ± 0.405	25
Thr	0.824 ± 0.014	39.603 ± 1.000	48
Arg	7.185 ± 0.054	45.768 ± 1.197	6
Ala	7.156 ± 0.083	141.764 ± 1.410	20
Tyr	2.986 ± 0.051	36.48 ± 0.583	12
Cys	ND	39.609 ± 8.261	*
Val/Met	3.198 ± 0.053	23.171 ± 0.220	7
Trp	5.636 ± 0.091	10.911 ± 0.206	2
Phe	5.79 ± 0.065	15.497 ± 0.400	3
Ile	1.312 ± 0.011	8.463 ± 0.077	6
Leu	3.151 ± 0.058	14.406 ± 0.228	5
Lys	1.815 ± 0.229	23.569 ± 2.540	13
Pro	2.117 ± 0.163	16.665 ± 0.682	8
Overall amino acids	58.812 ± 0.376	1481.286 ± 10.331	25

* indicates that the amino acid was not present in the unfermented sample and was present in the fermented sample.

**Table 3:** Ingredient composition of diets (g/100 g).

	ND	HD + CON	HD + UO	HD + FO
Normal diet	100	0	0	0
High-fat diet	0	80	80	80
Starch	0	4.8	0	0
Casein	0	11.5	0	0
Corn oil	0	3.7	0	0
Unfermented okara	0	0	20	0
Fermented okara with *Aspergillus* spp.	0	0	0	20
Energy (kcal/100 g)	359	432	428	436

**Table 4:** The macronutrient composition of unfermented okara and okara fermented with *Aspergillus* spp. (g/100 g).

	Unfermented Okara (UO)	Fermented Okara with *Aspergillus* spp. (FO)
Carbohydrates	10.9	16
Protein	26.29	36.2
Total Dietary Fibre	61.37	38.13
Fat	10.9	8.88
Total calorie (kcal/100 g)	246.86	288.72

## Data Availability

The data presented in this study are available on request from the corresponding author. The data are not publicly available due to patent preparation.
